# The TSH/Thyroid Hormones Axis and Breast Cancer

**DOI:** 10.3390/jcm11030687

**Published:** 2022-01-28

**Authors:** Ioannis A. Voutsadakis

**Affiliations:** 1 Algoma District Cancer Program, Sault Area Hospital, Sault Ste. Marie, ON P6B 0A8, Canada; ivoutsadakis@yahoo.com or ivoutsadakis@nosm.ca; 2 Section of Internal Medicine, Division of Clinical Sciences, Northern Ontario School of Medicine, Sudbury, ON P6B 0A8, Canada

**Keywords:** thyroid hormones, thyrotropin, DIO, breast cancer, prognosis, TRα, TRβ

## Abstract

Breast cancer, the most prevalent female carcinoma, is characterized by the expression of steroid nuclear receptors in a subset of cases. The most important nuclear receptor with prognostic and therapeutic implications is the Estrogen Receptor (ER), which is expressed in about three out of four breast cancers. The Progesterone Receptor (PR) and the Androgen Receptor (AR) are also commonly expressed. Moreover, non-steroid nuclear receptors, including the vitamin D receptor (VDR) and the thyroid receptors (TRs), are also present in breast cancers and have pathophysiologic implications. Circulating thyroid hormones may influence breast cancer risk and breast cancer cell survival, through ligating their canonical receptors TRα and TRβ but also through additional membrane receptors that are expressed in breast cancer. The expression of TR subtypes and their respective isotypes have diverse effects in breast cancers through co-operation with ER and influence on other cancer-associated pathways. Other components of the TSH/thyroid hormone axis, such as TSH and selenoiodinase enzymes, have putative effects in breast cancer pathophysiology. This paper reviews the pathophysiologic and prognostic implications of the thyroid axis in breast cancer and provides a brief therapeutic perspective.

## 1. Introduction

Breast cancer is a major cause of morbidity and mortality in women across geographies and age groups. It represents the top cancer in terms of prevalence and in mortality among women globally [1]. The majority of breast cancers express the Estrogen Receptor (ER) and display ER dependence for their growth. Thus, targeted therapies inhibiting the ER or inhibiting production of the main ligand estradiol have been the mainstay of their treatment [2]. Other steroidal Nuclear Receptors (NRs), such as the Progesterone Receptor (PR) and the Androgen Receptor (AR), play a role in breast cancer. For example, PR, as a target of ER transcription, is an indicator of ER functional status, but also has additional functions in its own right. AR is an alternative functional receptor, expressed in a subset of cancers, either with or without ER expression [3]. Non-steroidal NRs, such as vitamin D Receptor (VDR) and nuclear receptors for thyroid hormone (TRs), are also expressed in breast cancer and play functional roles in breast cancer cells, although these roles are less critical than that of ER. VDR is a regulator of calcium metabolism in kidney, bones and intestine [4]. It is also expressed in mammary tissues and breast cancers, where it suppresses proliferation and promotes apoptosis in established cancers [5]. Low circulating levels of the VDR ligand, vitamin D, are commonly associated with breast cancer development [6]. Furthermore, thyroid hormone receptors are expressed in a subset of breast cancers and influence cancer cell survival.

Thyroid hormones, thyroxine (T4, also called tetra-iodothyronine) and triiodothyronine (T3) are produced by the thyroid gland after receiving signals through the pituitary hormone Thyroid Stimulating Hormone (TSH, also called thyrotropin). TSH binds receptor TSHR in the surface of thyroid follicular cells. After secretion by the thyroid to the circulation, T4 is transformed to T3 inside target cells by deiodinases DIO1 and DIO2, which remove one of the iodine atoms of T4. Thyroid hormone receptors, TRα and TRβ, bind T3 in peripheral target tissues and exert multiple systemic effects. Thyroid hormones stimulate metabolic processes, increasing body heat production, and oxygen consumption as well as the uptake of cholesterol, triglycerides and glycose [7]. Thyroid hormones also increase blood supply in tissues by the vasodilation effect and lead to an increase in cardiac output by increasing both heart rate and contractility. Thyroid hormones collaborate with other hormones in the regulation of growth and fertility. Most tissues receive direct or indirect regulatory physiologic inputs from the thyroid cascade, which results in regulation of the expression of hundreds of genes.

This article reviews the role of the thyroid axis in breast cancer, with a focus on the circulating levels of TSH and T4 as well as on the expression and function of TRs and TSHR in breast cancer cells. Moreover, the effects of thyroid hormones mediated by non-nuclear receptors, which are present in breast cancers, are discussed. Breast cancer is a heterogeneous disease with at least four major sub-types, the clinical equivalents of genomic luminal A, luminal B, HER2 enriched and basal-like genotypes derived from genomic studies [8,9]. The expression of thyroid receptors and the pathophysiologic implications of the thyroid axis in these sub-types may be diverse and are also considered.

## 2. The Hypothalamic–Pituitary–Thyroid Axis and Systemic Effects Related to Cancer Pathogenesis

**Figure 1:**
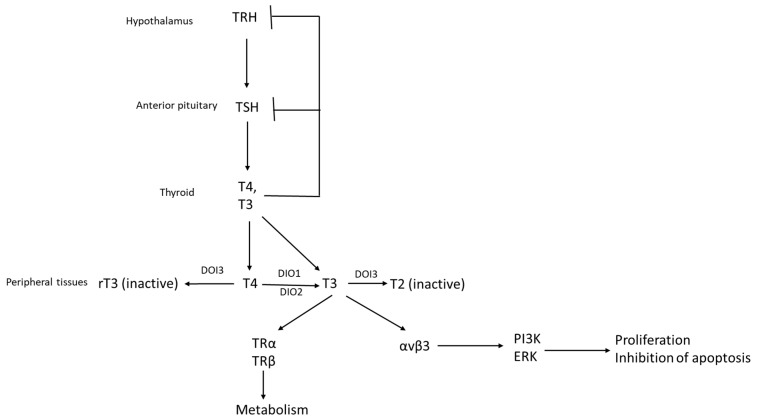
The TRH/TSH/Thyroid hormone axis. For details, please see text. TSH, Thyroid Stimulating Hormone; TRH, Thyrotropin Releasing Hormone.

Production of the glycoprotein hormone TSH by the hypophysis is stimulated by Thyrotropin Releasing Hormone (TRH), produced by neurons of the paraventricular nucleus of the hypothalamus [10]. TRH is a neuropeptide consisting of pyro-glycine, histidine and proline and is a positive regulator of the expression of the *TSHB* gene encoding for the beta subunit of TSH. TSH is a heterodimer and consists of an alpha and a beta polypeptide. The alpha sub-unit is shared with other pituitary hormones, Follicle Stimulating Hormone (FSH), Luteinizing Hormone (LH) and human chorionic gonadotropin (hCG) and the beta sub-unit is specific for TSH and specifies interaction with the TSH Receptor (TSHR). The glycosylation of TSH affects its potency and is also regulated by TRH [11]. TSH enters the circulation and reaches the thyroid gland, where it binds TSHR in the basolateral membrane of thyroid follicular cells and stimulates iodine mobilization and the production of thyroid hormones T4 and T3 (Figure 1) [12]. T3 acts in target tissues through the ligation of nuclear receptors, TRα and TRβ, which then function as transcription factors executing a metabolic program. Moreover, T3 and T4 have non-genomic actions through binding to cell surface integrin αvβ3 [13]. The specific DNA binding sequences of TRs, called TR Response Elements (TREs), consist of the AGGTCA sequence as a half site, which is repeated in a direct, everted, or inverted manner in different promoters [14,15]. T4 is the main circulating hormone and is de-iodinized to T3 in target tissues by iodothyronine deiodinases DIO1 and DIO2 [16]. Another deiodinase, DIO3, further deiodinizes T3 and T4, producing the inactive forms T2 and reverse T3 (rT3), respectively. T3 and T4 also exert a feedback action in anterior pituitary thyrotrope cells, shutting down the production of TSH, through negative regulations of the promoters of alpha and beta TSH subunits (Figure 1). They also act on hypothalamic neurons to decrease the production of TRH. Alpha subunit production is under the control of other hormones, such as gonadal steroids, and is increased in post-menopausal women. As a result, increased TSH levels may occur with normal aging and are not associated with hypothyroidism, except if concomitant thyroid pathologies, such as autoimmune thyroiditis, are present [17]. The feedback loops from circulating thyroid hormone levels, together with TRH, are the major regulators of physiologic TSH levels [17]. An excess of thyroid hormones (hyperthyroidism) leads to the suppression of TRH and TSH. Additionally, TSH is suppressed by exogenous steroids, by severe illness and by dopamine. In contrast, hypothyroidism causes the elevations of TSH [18]. TSH, especially when elevated, may produce direct effects in cells that express the receptor TSHR, independently of thyroid hormones.

TRα and TRβ receptors have overlapping but distinct target genes, and they have three and two isoforms, respectively, TRα1, TRα2 and TRα3 and TRβ1, and TRβ2, produced by alternative splicing [15]. The two TR receptors enter the nucleus after ligation by T3 and bind TRE sequences as either homodimers or heterodimers with the Retinoid X Receptor (RXR).

Target organs of thyroid hormones include the heart, the central nervous system, organs of the musculoskeletal apparatus and the kidney [18]. Genomic effects are mediated by regulation of target gene promoters bound by TRs ligated by T3. Non-genomic effects are mediated by the ligation of surface integrin αvβ3 (vitronectin receptor) and triggering of signal transduction pathways through kinases PI3K and ERK [13]. Target genes mediating genomic effects include direct targets of TRs and indirect targets that are transcribed by other transcription factors that are themselves direct targets of TRs. As an illustrative example, TRα directly regulates the CTNNB1 gene encoding for β-catenin, which then participates in the upregulation of the transcription of cyclin D and oncogene c-Myc [19,20]. Through these actions, TRs regulate cellular metabolism, proliferation and apoptosis [12].

The hypothalamic–pituitary–thyroid axis may directly affect cancer pathogenesis by affecting cancer cells when receptors for ligands of the axis are expressed in tumors, and indirectly affect cancer pathogenesis by altering systemic factors. An excess of thyroid hormones, as observed in clinical or sub-clinical hyperthyroidism, is associated with an increased incidence of various common cancers including prostate cancer, lung cancer, gastrointestinal cancers, and breast cancer [21,22]. Elevated thyroid hormone levels are associated with an increased incidence of breast cancer in diverse populations. For example, in a study from Brazil, patients with breast cancer had higher free T3 and T4 levels, and lower TSH levels, than age-matched controls [23]. In a population-based study from the United Kingdom, in post-menopausal women, the ratio of free T3 to estradiol was also significantly higher in breast cancer patients than in controls [24]. In contrast, this investigation disclosed no association of TSH with breast cancer risk and found an increased risk of breast cancer in patients with thyroiditis. Furthermore, in a study from Germany, breast cancer patients had higher free T3 and T4 levels than patients with benign breast tumors and healthy controls without breast pathologies [25]. In a study from the Swedish Cancer Registry, the risk of breast cancer was higher in women with higher levels of T3, in the higher quartile compared with women in the first quartile of T3 levels [26]. Women with high T3 levels more often had cancers of a larger size, with positive lymph nodes and negative expression of ER and PR [27]. Moreover, hyperthyroidism is often associated with adverse survival outcomes after cancer diagnosis. Conversely, primary hypothyroidism is associated with reduced breast cancer risk and more indolent cancers, as suggested by the smaller tumor size and lower rates of pathologic lymph node involvement [28]. Despite this reduced risk, hypothyroidism is associated with obesity, which is a risk factor for the development of ER-positive breast cancers [29]. Thus, hypothyroidism may influence the risk of breast cancer in difference directions through direct and indirect effects. The following sections examine the indirect and direct effects of the hypothalamic–pituitary–thyroid axis in breast cancer.

## 3. Expression and Actions of Thyroid Hormone Receptors and Other Proteins of the Axis on Breast Cancer Cells In Vitro and In Vivo

The effects of thyroid hormones have been examined in various experimental models of breast cancer, confirming the influence of the thyroid axis in cancer cell survival and proliferation. T3 stimulated proliferation of the hormone-sensitive breast cancer cell line T47D and led to the downregulation of tumor suppressors p53 and Retinoblastoma protein (Rb), in a manner similar to estradiol [30]. An estrogen receptor antagonist was able to reverse these effects of T3, suggesting that thyroid hormone effects are at least partially mediated through co-operation with ER in this model. The proliferation of both T47D cells and MCF7 cells was induced by T3 and estradiol in another study, which also showed that T3 was an inducer of an Estrogen Receptor Element (ERE), although it was significantly weaker than estradiol [31]. ER-positive breast cancer cell lines MCF7, T47D and ZR75 displayed a synergistic enhancement of growth following exposure to estradiol and T3, while this growth induction was not observed in ER-negative cell lines MDA-MB-231 and MDA-MB-468 [32]. The transfection of MDA-MB-231 cells with ER sensitized them to inhibition by estradiol and T3, an opposite effect to that observed in endogenously ER positive cells. The action of T3 on potentiation of the effects of estradiol in ER-positive cells requires TRs, as an RXR-selective retinoid ligand SR11217 that prevents the interaction of TRs with transcriptional partner RXR, and abrogates the synergistic effect of T3 to estradiol [30]. In a mouse model of 7,12-dimethylbenza-anthracene (DMBA)-induced breast carcinogenesis, euthyroid mice developed more voluminous tumors with a shorter latency than hypothyroid mice [33]. Other studies have shown that T3 stimulates the growth of MCF-7 cells through ER and induces the expression of ER target genes PR and EGFR [34]. This proliferative effect is not observed in the ER-negative cell line MDA-MB-231. However, in another study, the treatment of MCF-7 cells with T3 induced apoptosis by a mechanism involving the transcriptional suppression of apoptosis-negative regulator regucalcin (also called Senescence Marker Protein 30 (SMP30)) [35].

The specific type of TR expressed in a particular cell is critical for the genomic effects of thyroid hormones. Cell line MDA-MB-468 is negative for the expression of TRβ1. The transfection of MDA-MB-468 cells with TRβ1 led to a reduction in the growth of xenografts in mice in vivo compared with xenografts of non-transfected parental cells [36]. In addition, TRβ1-transfected MDA-MB-468 cells displayed decreased invasion and metastasis and underwent a partial mesenchymal-to-epithelial transition. These results were corroborated by another study in MDA-MB-468 cells transfected with TRβ [37]. After transfection, TRβ-expressing cells acquired a more epithelial-like phenotype, with a reduction in mesenchymal cytokeratins expression. TRβ1 transfection leads to the suppression of the expression of receptors EGFR1 and HER3, as well as IGF-IR [36]. The TRβ repressive complexes recruit co-repressors, such as Nuclear Receptor Co-repressor 1, (NcoR1) to silence the transcription of target genes, including receptor tyrosine kinase ligands VEGF-C and VEGF-D [38]. The VEGF axis is crucial for breast cancer lymphangiogenesis [38]. In TRβ expressing T47D, treatment with T3 suppressed the activity of STAT5 signal transducer, leading to the suppression of proliferation, whereas the expression of a mutant form of TRβ allowed for the de-repression of STAT5 and sustained proliferation [39]. TRβ, in co-operation with transcription partner RXRβ, directly represses the transcription of pro-tumorigenic β-catenin in thyroid and cervical cancer cells [40]. The phosphorylation of TRβ at tyrosine 406 seems to be a pre-requisite for its tumor-suppressing function [41]. Cells transfected with a TRβ mutated at position 406 (phenylalanine replacing the native tyrosine) showed a growth ability in rats that was equivalent to cells transfected with an empty vector, while xenografts of cells transfected with a wild-type TRβ were deficient in their growth.

The expression of αvβ3 integrin (also known as the vitronectin receptor) marks vascular beds associated with tumor neovascularization [42]. αvβ3 integrin can differentiate tumor-associated endothelia better than CD31, which is also expressed in normal endothelial cells. αvβ3 integrin is expressed in tumor vessel endothelial cells and tumor cells of breast cancer patients [43]. αvβ3 integrin expression is induced by Transforming Growth Factor beta (TGFβ) signaling in breast cancer cells [44]. αvβ3 integrin acts as a cellular receptor for the thyroid hormones, and its expression in breast cancer cells has an association with bone metastases in rats and in mice bearing human breast xenografts [45,46]. T47D cells exposed to T3 display activation of the FAK and AKT kinases through αvβ3 integrin binding, resulting in a remodelling of focal adhesion and the promotion of mobility and invasion [47]. The αvβ3-integrin-mediated activation of FAK kinase and the PI3K/AKT cascade may also be executed by lipid 14,15-epoxyeicosatrienoic acid in breast cancer cells and promotes EMT and cisplatin resistance [48]. The MAPK cascade is also activated by FAK and induces proliferation [49]. The high expression of αvβ3 integrin has been reported in triple-negative breast cancers with the mesenchymal phenotype [50]. An antagonistic peptide, called ψRGDechi, decreased the migration and invasion of mesenchymal breast cancer cells and their ability to form mammospheres, suggesting decreased stem cell properties. Tetraiodothyroacetic acid (tetrac), an antagonist of thyroid hormones, acts through the αvβ3 integrin to inhibit the proliferation of MDA-MB-231 cells [51]. Tetrac results in the downregulation of apoptosis inhibitors XIAP (X-linked inhibitor of Apoptosis) and MCL1 (Myeloid Cell Leukemia 1). These effects are maintained when cells are exposed to nanoparticulate tetrac, which is not able to penetrate the cytoplasm [51].

As well as actions carried out through αvβ3 integrin and downstream pathway activation, thyroid hormones have pleiotropic effects through thyroid receptors in cancer cells by co-operating with other pathways [52]. In the context of the bowel, TRs co-operate with β-catenin to promote proliferation in a pathway that is active in crypt stem cells and is strongly activated in colorectal cancers due to APC mutations [20]. Given that β-catenin, but also the soluble frizzled ligand sFRP2, are targets of thyroid receptor transcription, a positive activating loop exists between the thyroid and Wnt/β-catenin transcriptional programs in cells where the two programs are functional [53].

The systemic effects of circulating thyroid hormones on breast carcinogenesis were examined in a model of DMBA-induced breast carcinogenesis in rats [54]. Rats were exposed to DMBA and treated with propylthiouracil or T4 to become hypothyroid or hyperthyroid, respectively. Hypothyroid rats showed a lower incidence of mammary tumors, which developed later and had a lower tumor growth rate than tumors in euthyroid controls and hyperthyroid rats. However, these effects may have been indirect, given that hypothyroid rats displayed reduced serum levels of estradiol [54]. Other systemic effects of hypothyroidism observed in rats included a lower serum leptin level with preserved abdominal fat mass, while circulating progesterone and prolactin levels were not different compared with controls. In another in vivo animal model of immunocompetent Balb/c mice, which were inoculated with triple-negative 4T1 breast cancer cells, tumors in hyperthyroid mice grew faster than tumors of hypothyroid animals and displayed decreased infiltration by cytotoxic lymphocytes [55]. However, lung metastases were more numerous in hypothyroid animals compared with hyperthyroid animals.

Overall, data from animal and in vitro models confirm that the thyroid axis may have both pro-carcinogenic and tumor-suppressing effects. The specific thyroid nuclear receptor expressed is critical in mediating thyroid hormone effects and, in addition, αvβ3 integrin is expressed in breast cancer cells and is equally important for the observed effects. A model, consistent with the one for estrogens, where high levels of hormones promote mammary carcinogenesis but are associated with the development of more indolent disease, is suggested by in vivo models of thyroid-hormone-facilitated mammary carcinogenesis in mice. Among the receptors responding to thyroid hormones, TRα and αvβ3 integrin are pro-proliferative, while TRβ mainly has tumor-suppressing effects.

## 4. Expression of TRs, TSHR and Related Proteins in Human Breast Cancers and Prognosis According to Subtype

A direct effect of the thyroid axis can be anticipated in breast cancers that express receptors for the thyroid hormones, either the TRs or the αvβ3 integrin (Table 1). Thus, determination of the expression of these receptors in breast cancer cells is of particular interest. The expression of thyroid receptor TRα1 was reduced in breast cancers compared with normal, benign (fibrocystic changes and fibroadenomas) and lactating breast tissues [56]. TRα expression was even lower in more sizable and higher-grade breast cancers compared with smaller and lower-grade ones [57]. The cellular localization of TRα and TRβ was examined by immunohistochemistry in a study of 148 samples from normal breast tissues, benign breast lesions and in situ or invasive breast cancers [58]. TRα was expressed in nuclei of epithelial cells lining normal breast ducts but was excluded in the cytoplasm of cells from benign and malignant lesions. In infiltrating carcinomas, TRα expression displayed an inverse correlation with cell proliferation, as measured by PCNA (Proliferating Cells Nuclear Antigen). TRβ was cytoplasmic in benign ducts. Benign breast lesions and in situ carcinomas showed a nuclear localization of TRβ, while invasive carcinomas mostly showed cytoplasmic staining of the receptor [58]. In addition to sporadic breast cancers, the expression of TRα and TRβ was confirmed in breast cancers associated with *BRCA1* gene mutations [59]. TRα was positive in 41.9% of sporadic cancers and 44.7% of BRCA1-associated cancers in this study. TRβ was positive in 22.1% of sporadic cancers and 52.6% of BRCA1-associated cancers. In contrast to sporadic cancers, TR receptors had prognostic significance in BRCA1-associated cancers but reverse correlations between TRα and TRβ. BRCA1-associated cancers with positive TRα had an inferior prognosis compared to counterparts that were negative for TRα expression [59]. The reverse was true for TRβ, with BRCA1-associated cancers that were positive for TRβ expression having a better prognosis than TRβ-negative counterparts. Cells with *BRCA1* mutations were unable to degrade TRα in vitro, suggesting that dysregulated excess activity may contribute to adverse outcomes.

The expression of different isotypes of TRα and TRβ receptors in varying frequencies in breast cancer was confirmed in another study [60]. Isotypes TRα1 and TRα2 were expressed in about 70% of breast cancers, while TRβ1 receptors were expressed in 54% of cases, and TRβ2 was the most commonly expressed isotype in 79% of cases. No significant prognostic associations were observed, but a trend was noted for TRα2 and disease-free survival as well as overall survival. The Allred score was equal to or greater than 6 (TR Allred score of 6 or above was considered positive in this study) for TRα1 and TRα2 in 74% and 40% of breast cancer patients in another series [61]. TRα2 expression correlated with expressions of ER and PR, with HER2 negativity, and with improved 5-year OS [61]. However, another study showed no clear association between TRα expression and breast cancer intrinsic sub-types or hormonal profiles [57]. ER and TRs were concomitantly expressed in a study of breast cancer tissues from 12 patients [62]. The ex vivo exposure of breast cancer cells to T3 and estradiol showed that the two hormones had partial agonistic effects, initiating transcription from each other’s target promoters [63]. When exposed to T3, ex vivo breast cancer cells upregulated the expression of EGFR, an effect that was reversed by tamoxifen. High TRβ1 expression, as defined by an Allred score above 4, was associated with a longer breast-cancer-specific survival compared with low TRβ1 expression in women with localized breast cancer [64]. In this extensive database study, 40% of patients (318 of 796 of patients) had a high TRβ expression, with an Allred score above 4. TRβ positivity with this cut-off was observed in 92.4% of ER-positive tumors and in 68% of ER-negative tumors [64]. In another study, specifically focusing on triple-negative breast cancer patients, lower TRβ expression was associated with worse outcomes [63]. In vitro evaluation with TRβ knockdown suggested that the tumor-suppressing effects of TRβ were mediated by the suppression of cAMP/Protein Kinase A (PKA) signalling [65].

**Table 1:** Studies of thyroid receptors in breast cancers discussed in this article.

Study	Number of Patients	Type of Cancer	Receptor Sub-Type	Findings
Alyusuf et al. [54]	46 patients with cancer and 100 controls with benign lesions and normal tissues	All	TRα1	Decreased expression in cancer compared to normal mammary tissue and benign lesions.
Charalampoudis et al. [55]	41 patients (tumors and normal tissues)	All (23 ER-positive, 11 HER2-positive)	TRα	Decreased expression in cancer compared to normal mammary tissue. Partial loss in larger and high-grade tumors.
Conde et al. [56]	52 patients with invasive cancers, 20 patients with in situ cancers and 12 controls with benign lesions	All	TRα, TRβ	TRα located in nuclei of normal cells and the cytoplasm of pathologic lesions. The reverse is true for TRβ except for invasive cancers, which showed a similar cytoplasmic localization to TRα.
Heublein et al. [57]	86 sporadic and 38 BRCA1-associated breast cancer patients	All	TRα, TRβ	TRβ is a good prognostic factor in BRCA1-associated cancers and TRα is an adverse prognostic factor.
Ditsch et al. [58]	82 patients	All	TRα1, TRα2, TRβ1, TRβ2	Both receptor isotypes are expressed in subsets of breast cancers.
Jerzak et al. [59]	130 patient	All (95 ER-positive, 17 HER2 positive)	TRα1, TRα2	TRα1 and TRα2 positivity (Allred score above 6) was observed in 74% and 40% of cases.
Jerzak et al. [62]	796 patients	All (616 ER-positive, 219 HER2-positive)	TRβ1	High expression of TRβ1 is associated with better cancer-specific survival.
Gu et al. [63]	227 patients	Triple-negative breast cancers	TRβ	High expression of TRβ1 is associated with better disease-free survival

The expression of αvβ3 integrin receptor facilitates the metastatic process, and especially the early steps of metastasis [66]. The expression of αvβ3 integrin was associated with adverse disease-free survival outcomes in triple-negative breast cancers, with high expression (above the median of the group) of the integrin receptor [67].

TSHR is a protein of 87 kDa and belongs to the family of G-coupled protein receptors (GCPRs) [68]. The activation of TSHR in the plasma cell membrane transmits signals through G proteins Gas and Gaq, and downstream through the cAMP/PKA/CREB pathway and the phospholipase C/PI3K/AKT/mTOR pathway. The role of TSHR is established in well-differentiated thyroid cancers, where treatment with T4 to suppress TSH secretion from the hypophysis, in order to prevent TSHR activation, is the mainstay of treatment [69]. TSHR is also expressed in other cancers, such as ovarian carcinomas, hepatomas and melanomas [68,70,71,72]. The expression of TSHR is increased in breast cancer tissues compared to adjacent normal mammary tissues [73]. However, the functional significance of the increased expression of TSHR in breast cancer is unclear. Specifically, the effects, if any, of direct TSH signaling through TSHR expressed in breast cancer cells have not been confirmed.

The deiodinases (DIOs) are an integral part of the TSH/thyroid hormone axis and can regulate the actions of the axis through the production and disposition of thyroid hormones. DIO3 is expressed in the normal mammary luminal duct and acini cells and, at similar levels, in breast carcinomas [74]. There was no difference in DIO3 expression between cancers with positive or negative ER and positive or negative HER2 receptors. Patients with higher DIO3 levels (above the median expression by immunohistochemistry) had a better survival than patients with DIO3 expression below the median of the group [74]. A similar difference in survival was observed when breast cancer patients from the TCGA cohort were categorized according to their DIO3 mRNA expression levels. DIO1 was not differentially expressed between normal mammary tissues, benign breast lesions and breast cancers [56]. In contrast, another study found increased mRNA expression and enzymatic activity in breast cancers compared with surrounding normal tissues [75]. DIO1 was expressed in ER-positive cell line MCF-7 but not in ER-negative cell line MDA-MB-231 [76].

Selenium is a trace element that facilitates the function of the family of selenoproteins such as deiodinases. Selenium levels, as measured in plasma and toenails, have been associated with breast cancer risk, suggesting that the function of thyroid-hormone-metabolizing enzymes are involved in aspects of breast cancer pathogenesis [77].

The transcriptional corepressor Silencing Mediator of Retinoid and Thyroid Receptors (SMRT) plays a role in gene repression by nuclear receptors that are not bound by their respective ligands. In a series of breast cancers, higher SMRT expression by immunohistochemistry was associated with lower circulating levels of T3 and a higher tumor grade and proliferating index, as measured by the marker Ki-67 [78]. The overexpression of SMRT and lower levels of circulating TSH (below the median in the group) were associated with adverse survival outcomes in this study [78].

In conclusion, studies of thyroid hormone receptors and related proteins in breast cancer tissues show that the TR subtypes are expressed in most breast cancers. Their localization is altered compared to normal mammary epithelia and they tend to be excluded to the cytoplasm, where they are not transcriptionally active. TRβ expression is particularly associated with improved survival. The expression and function of other components of the thyroid axis in breast cancer are less studied, and their role remains less clear.

## 5. Circulating Thyroid Hormones and Breast Cancer Prognosis

An association of thyroid function with survival outcomes in patients diagnosed with breast cancer has been suggested in diverse populations, as discussed in a previous section. TSH and thyroid hormone levels after the diagnosis of breast cancers are more informative for the care of breast cancer patients, as they could have prognostic implications in these patients. The contrasting effects of the thyroid axis on the risk of developing breast cancer and prognosis of established breast cancers may underpin the results of thyroid hormones studies in breast cancer patients presented in the following paragraphs, which are in contrast to the results of thyroid hormones as risk factors for breast cancer in the general population.

A subgroup of 551 patients who were diagnosed with breast cancer from the Malmö Diet and Cancer Registry were analyzed for breast cancer outcomes according to levels of circulating thyroid hormones and TSH [79]. Levels of free T4 in the second (mid) tertile of the group were associated with statistically significant better breast cancer mortality compared with the low tertile (hazard ratio 0.50, 95% confidence interval 0.29–0.86). Patients with the high tertile of T4 also showed a trend of improved breast cancer mortality, although this was not statistically significant. Neither free T3 levels nor TSH levels were associated with mortality [79]. Consistent with these results, in another study, low circulating levels of T4, but also of T3, as well as a high TSH, were associated with a more advanced stage of breast cancer and disease with positive lymph node and metastases [80]. At least part of the thyroid hormone association with breast cancer prognosis is explained by differences in the functional status of the thyroid axis in breast cancer sub-types. High serum levels of T4 were observed in women who mostly developed ER- and PR-positive breast cancers in the Malmö Diet and Cancer Registry [81]. In another, smaller study of 97 women newly diagnosed with breast cancer and healthy controls with negative mammograms, T4 levels were higher in breast cancer patients compared with controls [82]. In contrast, baseline levels of T3 and TSH did not differ between breast cancer patients and controls. These data regarding circulating thyroid hormones in conjunction with the functional significance of the expression of thyroid receptors in breast cancer, as discussed in the previous section, are reminiscent of the estrogen/ER axis association with the promotion of established breast cancers but decreased malignant potential in ER positive cancers.

Possibly, at odds with the above data, in a retrospective cohort of 437 breast cancer patients categorized according to ER, PR and HER2 receptors, higher levels of TSH were associated with hormone-receptor-positive/HER2-negative cancers compared with hormone-receptor-positive/HER2-positive cancers. No other associations of TSH levels with sub-types or pathologic features of aggressiveness were discerned [83]. In addition, whether higher TSH levels was associated with decreased circulating thyroid hormones in this cohort was not reported. If, indeed, higher TSH levels denote subclinical hypothyroidism, these data suggest that hormone-receptor-positive/HER2-positive cancer patients are more commonly euthyroid than patients with hormone-receptor-positive/HER2-negative cancers. This would support the role of thyroid axis perturbations in the pathogenesis of luminal breast cancers but not in HER2-dependent disease.

## 6. Therapeutic Perspective

Steroid nuclear receptors have a well-established role in endocrine-dependent cancers such as breast and prostate. The inhibition of estrogen receptors or suppression of their ligand levels are therapeutic manipulations with proven efficacy in ER-positive breast cancers. Similarly, thyroid hormones and thyroid receptors play a role in the treatment of thyroid cancers. However, despite the expression of thyroid receptors in a variety of tissues and cancers, their role in other cancers remain less well-defined. Studies of TRα and TRβ expression in breast cancer have shown their presence in most breast cancers and have suggested an association with improved breast cancer outcomes, especially for TRβ. Data regarding the expression and influence of other components of the thyroid hormone axis in breast cancer are sparser. Despite experimental confirmation of a pro-carcinogenic role of the thyroid membrane receptor αvβ3 integrin in cancer, few data exist on the influence of thyroid hormones through the ligation of this receptor on breast cancer. Conceivably, the influence of the αvβ3 integrin in breast cancer cells extends beyond its function as a membrane thyroid hormone receptor and is mediated through other ligands of the extracellular matrix, such as the canonical ligand vitronectin [84]. αvβ3 integrin has been studied as a targeting molecule of therapeutic delivery in cancer cells that express the receptor [85]. A similar approach to blocking the αvβ3 integrin receptor with the goal of interfering with thyroid hormone effects in breast cancers could be envisioned. This approach would have the additional benefit of maintaining the potential anticarcinogenic effects of thyroid hormones mediated by TRs.

Several additional factors may contribute to the lack of a clearer picture regarding thyroid hormones and the pathogenesis of breast cancer. One factor is the close interrelationships of thyroid hormone effects in breast tissues with the effects of female hormones. For example, the effect of a longer exposure to sex hormones due to longer ovulatory activity, a classic breast cancer risk factor, was more pronounced in patients with lower circulating T4 levels [86]. In addition, some of the effects of TRs in breast cancer cells that were observed in experimental models are only present in ER-positive cells, suggesting that the expression of ER modulates the function of TRs. It is conceivable that the thyroid axis actions vary according to the breast cancer subtypes, given the fundamental molecular differences underpinned by the expression of ER. Future studies examining the thyroid axis in each breast cancer subtype are needed to obtain a clear understanding. This would be a prerequisite for devising and developing therapeutic strategies that will exploit the manipulation of this axis in patient subsets with the highest probability of receiving any benefit. The degree of thyroid receptor expression varies between individual breast cancers and would have to be taken into consideration using the Allred score or a similar metric in immunohistochemistry section evaluations. Finally, another strategy to deconvolute the thyroid hormone receptor effect in breast cancer would involve the development of specific receptor activators for TRβ or inhibitors of the thyroid receptor site of αvβ3 integrin.

## 7. Conclusions

The thyroid axis represents a potential, unexplored target for the treatment of breast cancers. Both nuclear and cytoplasmic membrane receptors of the thryroid axis are expressed in a majority of breast cancer cells and would be rational targets. However, a nuanced approach, considering factors such as level of expression and the specific subtype of receptors that are expressed, as well as the molecular subtype of the breast cancer, is necessary to maximize the chance of developing a successful treatment.
